# Modified technique for resection of the alar folds to nasal paralysis in a mule: Case report

**DOI:** 10.1007/s11259-026-11527-3

**Published:** 2026-09-16

**Authors:** Marcos Jun Watanabe, Juliana de Moura Alonso, Pyetra Ieger Perandré, Heitor Cestari, Vitória Fabbrocini Gonçalves, Lorena Cardozo Ferrari, Ana Liz Garcia Alves, Carlos Alberto Hussni

**Affiliations:** 1 School of Veterinary Medicine and Animal Science, São Paulo State University (UNESP), Botucatu, SP Brazil; 2 Federal University of Rio Grande do Sul, Porto Alegre, RS Brazil

**Keywords:** Nasal collapse, Alar cartilage, Facial nerve

## Abstract

Equids are obligate nasal breathers. Therefore, conditions that obstruct or impair this respiratory route can compromise animal welfare by causing severe respiratory distress, while also impacting athletic potential. Nasal paralysis results from injury to the facial nerve and its branches and is characterized by the lack of movement of the alar cartilage, leading to nasal collapse. Its occurrence is uncommon in horses and has not been previously reported in mules. This report describes a 9-month-old mule presented with a history of facial trauma and compression caused by a halter. After unsuccessful anti-inflammatory therapy and temporary retention sutures, resection of the alar cartilage was elected. The procedure was performed under general anesthesia with the patient positioned in sternal recumbency. Traction was applied to the apex of the alar cartilage lamina, followed by an elliptical skin incision. Upon exposure, the alar cartilage was resected, and the skin was closed using a simple continuous suture pattern. The patient showed satisfactory recovery, with maintenance of airway patency. Two months postoperatively, the animal remained stable with no signs of nasal collapse. The investigation of therapeutic options and the description of surgical alternatives for correcting nasal collapse are fundamental, as nasal paralysis, although uncommon, can lead to severe dyspnea, result in nasal collapse, and compromise both animal welfare and athletic performance.

**Supplementary Information:**

The online version contains supplementary material available at https://doi.org/10.1007/s11259-026-11527-3.

## Introduction

Equids are obligate nasal breathers due to the complex anatomical configuration of their upper respiratory tract, particularly the close relationship between the elongated soft palate, intrapharyngeal ostium, epiglottis, pharynx, and larynx, which prevents oral breathing during inspiration. This mechanism is crucial for regulating the temperature and humidity of inspired air, as well as preventing the entry of foreign particles. This characteristic also limits the horse’s ability to switch to oral breathing when there is high nasal resistance, such as during intense exercise. While humans and dogs can alternate between nasal and oral breathing, horses depend exclusively on nasal breathing due to their specific anatomy (Legorreta 2006).

The alar folds are mucocutaneous structures located dorsally and rostrally within the equine nasal cavities. These folds create a separation between the true nostril and a small superior diverticulum known as the false nostril. The alar cartilage is an extension of the extension of the ventral nasal concha cartilage and extends caudally (Kallmyr et al. 2020).

During inspiration, the contraction of the *transversus nasi* muscle occurs, elevating the alar cartilage and resulting in the closure of the false nostril opening (Kallmyr et al. 2020). This mechanism helps regulate airflow during respiration, preferentially directing it to the true nostril and assisting in the filtration and humidification of inspired air. These adaptations are vital for the respiratory health and performance of equids (Strand et al. 2019).

Alar fold collapse is a condition that can occur when there is excessive size of the alar fold or failure in muscular action, especially of the transversus nasi. During collapse, air enters the false nostrils, causing flaccidity in the alar fold and partial obstruction of the nasal passages (Schumacher and Dixon 2007). Partial obstruction of the nasal passages can result in dyspnea, particularly during physical activity or stress (Strand et al. 2019).

The abduction movement of the alar cartilages resulting from the contraction of the transversus nasi muscle during inspiration is coordinated by the facial nerve and its branches. Trauma to the head region can lead to nerve injuries and, uncommonly, result in nostril paralysis with consequent alar fold collapse (Strand et al. 2019). The clinical manifestation of this condition is characterized by a vibratory noise or continuous humming during respiration, present during both expiration and inspiration. This condition is frequently associated with high expiratory pressures in the nasopharynx. Early diagnosis and treatment of alar fold collapse are essential to ensure the welfare and athletic performance of horses (Franklin and Allen 2017).

Surgical resection of the alar fold can be an effective therapeutic option to alleviate symptoms and improve respiratory function (Morris and Seeherman 1991; Martin et al. 2000). Traditionally, surgical resection is performed under general anesthesia with access through the external region of the nasal cavity, with or without the placement of a nasal prosthesis (Hawkins et al. 1995; Torre 2010; Strand et al. 2019). More recently, eight cases were described where alar fold and cartilage resection were successfully performed in a standing position under sedation, using the natural orifice and electrosurgery (Kallmyr et al. 2020).

The objective of the present report is to describe the treatment of nasal collapse resulting from a traumatic injury to the facial nerve in a mule, using a modified alar cartilage lamina resection procedure through the natural nostril orifice, using conventional hemostasis under general anesthesia.

### Case report

A 9-month-old mixed-breed mule colt was referred to the Veterinary Hospital with a complaint of severe respiratory distress and intense noise. The history described a facial trauma resulting from a halter accident approximately 90 days prior. During the trauma, there was compression of the face in the rostral region, facial crest, and nasal bone. Immediately after the trauma, dyspnea and respiratory noise were observed, along with nostril collapse during inspiration.

At the property, the referring veterinarian performed skin sutures on the dorsal aspect of the nostril in an attempt to maintain airway opening; however, dehiscence occurred, and the animal was referred for hospital care.

**Fig. 1:**
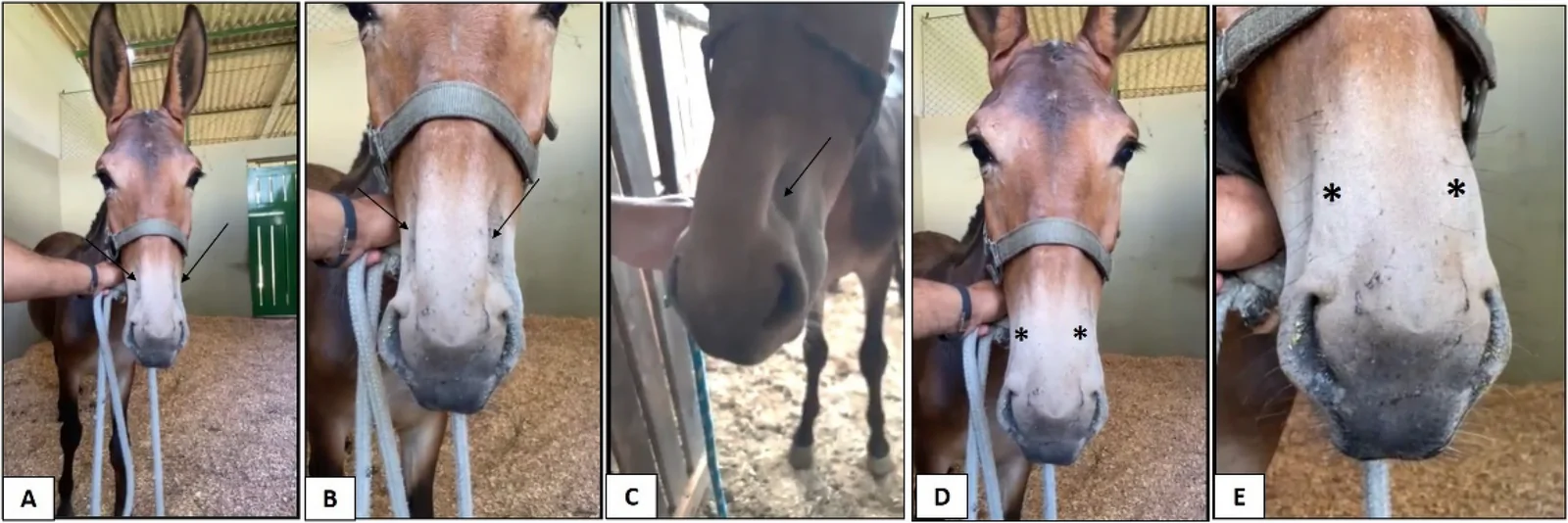
A 9-month-old mule exhibiting clinical signs of nasal paralysis. **A**, **B**, **C**. Presence of nostril collapse during inspiration; the arrows indicate the collapsed nostril skin and the failure of the false nostril to close, obstructing airflow into the nasal cavity. **D**, **E**. Absence of collapse during expiration; the asterisk (*) highlights the absence of skin collapsing into the nasal cavity

Upon admission, physical examination with the animal at rest revealed a decreased pain sensitivity in the rostral face, absence of alar cartilage abduction, and bilateral nostril collapse during inspiration, characterizing nostril paralysis (Video 1) (Fig. 1). Therapy was initiated with anti-inflammatories (meloxicam 2%, 0.6 mg/kg, IV SID), treatment of the facial nerve region (diethylammonium diclofenac 11.6% ointment, local massage), and a new suture was performed in a mattress pattern using USP 2 nylon. The suture was positioned on the dorsal aspect of the nostril and fixed under tension, pulling the alar cartilages dorsally. During the maintenance of the suture, there was an improvement in the respiratory pattern; however, dehiscence occurred after two days.

**Fig. 2:**
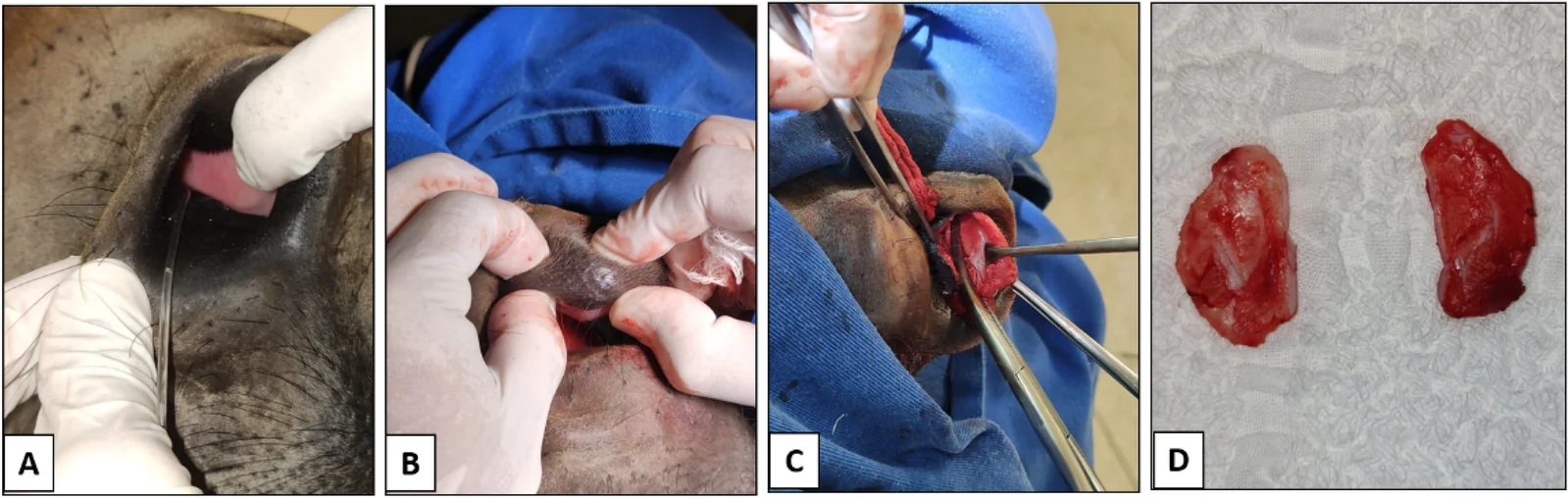
Modified surgical procedure for alar cartilage resection. (**A**) Confirmation of the nasolacrimal duct opening position; (**B**) Delimitation of the alar cartilage by palpation; (**C**) Clamping of the cartilage after skin incision and blunt tissue dissection; (**D**) Excised cartilages

Due to suture dehiscence, the risk of respiratory distress, and the prolonged clinical course, surgical treatment was elected, aiming for partial resection of the alar cartilages. The procedure was performed under general anesthesia. Premedication was performed with detomidine (0.02 mg/kg, IV), followed by anesthetic induction with ketamine (2.2 mg/kg, IV) and diazepam (0.05 mg/kg, IV). The patient was endotracheally intubated, and anesthesia was maintained with isoflurane in 100% oxygen. Sternal recumbency was chosen to maintain natural head symmetry, allowing direct, simultaneous visual assessment of both nostrils and ensuring optimal positioning for catheterizing the elevated nasolacrimal duct orifice unique to mules. Following retrograde catheterization of the nasolacrimal duct, clipping, and antisepsis of the nostril region, traction was applied to the apex of the alar cartilage lamina using an Allis fórceps (Fig. 2). An elliptical skin incision was made to enlarge the nasal ostium post-suturing. After exposing the alar cartilage lamina, curved Rochester forceps were applied to isolate its insertion at the ventral concha. Resection was performed by incising the cartilage adjacent to the forceps. Subsequently, the skin was sutured with 0 USP nylon in a simple continuous pattern (healed *per primam*). The procedure was then repeated on the contralateral nostril.

Postoperatively, the surgical technique proved effective in maintaining nasal cavity patency. After surgery, the patient received the following medications: ceftiofur (5 mg/kg IV SID for 7 days), flunixin meglumine (1.1 mg/kg IV SID for 3 days), and tetanus antitoxin (10,000 IU, single dose). Daily local wound care was performed with 0.12% aqueous chlorhexidine solution. After five days, the animal remained stable and was discharged. No improvement in facial sensivity was observed. The full course of antimicrobial therapy was completed at the farm by the local veterinarian. A follow-up telephone call two months post-surgery revealed that the animal remained stable with no signs of nasal collapse (Video 2).

## Discussion

The modified alar cartilage resection technique presented in this report successfully restored nasal airway patency and resolved clinical signs of dyspnea and respiratory noise in a 9-month-old mule with bilateral nasal collapse. Surgical resolution was achieved through a natural orifice approach under general inhalation anesthesia without specialized electrosurgical equipment. Primary wound healing was achieved without complications, and the animal remained asymptomatic at two months post-surgery.

The clinical signs of nasal collapse, combined with a history of facial trauma, supported the diagnosis of nasal paralysis secondary to injury to the buccal branch of the facial nerve, leading to impaired contraction of the transversus nasi muscle and subsequent failure to open the nasal cavity (Schumacher and Dixon 2007; Kallmyr et al. 2020). Diagnosis was confirmed clinically by temporary retention suturing, which temporarily abducted the alar cartilages and relieved respiratory noise (Beard 1996).

Concurrently, anti-inflammatory therapy was used in an attempt to restore nerve function, initially at the property and later at the hospital. The initial treatment goal was to maintain airway patency via nasal sutures while promoting nerve recovery through anti-inflammatories and physical therapy. However, repeated suture dehiscence, coupled with the lack of motor function recovery after more than 90 days of injury, the bilateral occurrence of the condition, and the risk of respiratory distress, led to the choice of surgical treatment to ensure airflow to the nasal cavity.

Since surgical access to the alar cartilage is typically indicated via an incision in the roof of the nostril (Auer et al. 2019), correct anatomical positioning is particularly critical in mules due to specific upper respiratory variations. Unlike horses, in which the nasolacrimal duct opens on the floor of the nasal vestibule, in mules it drains into the upper third of the lateral nasal wing (Wissdorf et al. 2021). Consequently, retrograde catheterization of the nasolacrimal duct prior to mucosal incision is essential in this species to prevent accidental transection or iatrogenic trauma during alar cartilage dissection.

Several surgical approaches for correcting nasal collapse have been described, including alar fold resection via external nasal access under general anesthesia (Hawkins et al. 1995; Strand et al. 2019), skin fragment excision from the dorsal nasal intake in standing horses (Kol and Kelmer 2011), insertion of rigid cartilaginous or metallic prostheses (Torre 2010), and complete resection via the natural orifice using bipolar electrosurgery in standing horses (Kallmyr et al. 2020). Permanent tracheostomy is considered a salvage procedure due to its high maintenance demands and cosmetic impact (Kallmyr et al. 2020).

The choice of general inhalation anesthesia and sternal recumbency was another deliberate technical modification. While natural orifice resection has been reported under standing sedation in calm horses (Kallmyr et al. 2020), general anesthesia was elected due to the young age and reactive temperament of the mule, ensuring patient safety and precise surgical maneuvering. Sternal recumbency was specifically selected to maintain natural bilateral head symmetry and anatomical alignment without facial distortion caused by lateral or dorsal recumbency. This symmetrical orientation facilitated simultaneous bilateral access, accurate elliptical skin incisions, and precise identification of anatomical landmarks.

In the present case, a modified natural orifice approach was performed, adapting the technique described by Kallmyr et al. (2020). A key modification was performing the resection using conventional sharp dissection and standard hemostasis rather than specialized bipolar electrosurgical devices (e.g., LigaSure). The lack of excessive intraoperative bleeding demonstrated that conventional hemostasis is sufficient for this procedure, making it accessible in clinical settings where advanced electrosurgical equipment is unavailable. Additionally, an elliptical skin incision with partial skin fragment removal from the alar fold was incorporated to enlarge and stabilize the nasal ostium post-suturing. While external nasal resection (Hawkins et al. 1995; Strand et al. 2019) and prosthetic support (Torre 2010) yield favorable outcomes, natural orifice access eliminates external facial scars and reduces recovery times. Prosthetic techniques also carry risks of donor site morbidity or implant rejection. The modified natural orifice technique described here provides a less invasive, cosmetically superior alternative while avoiding complex instrumentation.

This report has limitations that should be acknowledged. First, it describes a single case in a non-performance, young mule, limiting the broad generalizability of the findings to working or athletic equids. Second, the follow-up period was limited to two months postoperatively, and patient evaluation relied on clinical observations and owner reports rather than objective diagnostic tools such as upper airway endoscopy or airflow mechanics measurements. Finally, while the clinical outcome was successful without complications, a single case report cannot establish superiority over conventional surgical approaches. Further studies involving larger cohorts and long-term objective evaluations are warranted.

## Conclusion

The modified resection technique of the alar cartilage lamina was sufficient to prevent nasal cavity collapse and did not result in excessive bleeding, dyspnea, or short- to medium-term complications.

## Supplementary Information

Below is the link to the electronic supplementary material.

Supplementary Material 1

Supplementary Material 2

## Data Availability

No datasets were generated or analysed during the current study.
